# Increased soil nutrition and decreased light intensity drive species loss after eight years grassland enclosures

**DOI:** 10.1038/srep44525

**Published:** 2017-03-27

**Authors:** Jingpeng Li, Zhirong Zheng, Hongtao Xie, Nianxi Zhao, Yubao Gao

**Affiliations:** 1 College of Life Sciences, Nankai University, Tianjin, 300071, China; 2 State Environmental Protection Key Laboratory of Regional Eco-process and Function Assessment, State Key Laboratory of Environmental Criteria and Risk Assessment, Chinese Research Academy of Environmental Sciences, Beijing, 100012, China

## Abstract

Enclosures (fenced, grazing or clipping) within a certain period of years are the most common tools for restoration of degraded grasslands in temperate regions. Short-term enclosures can improve biodiversity and productivity by effectively relieving grazing pressure, while long-term enclosures can reduce species diversity. We therefore carried out a field experiment to investigate the specific causes of the reduced species diversity in Hulunbeier grassland of northern China. After eight years of enclosure, the significantly increased soil available nitrogen (AN) and available phosphorus (AvP) in enclosure community reduced nitrogen (N) limitation but most vegetation was still N limited. Many environmental factors led to decreased species richness, but increased soil AN and decreased light intensity at the community bottom were the most significant ones. Community density decreased independently of soil nutrition but significantly related to decreased species richness. Density of dominant canopy species increased, while dominant understory species decreased during assemblage-level thinning; therefore, the random-loss hypothesis was not supported. The dominant understory species responded to lower light availability by increasing their height, leaf area, and chlorophyll content. Moreover, our results were expected to provide some specific guidance for the restoration mode selection of degraded grasslands in northern China.

Northern China has experienced widespread grassland degradation as grazing pressure has grown to unsustainable levels. Grasslands function as an important ecological barrier, but their degradation reduces sustainable livestock production and unbalances the ecosystem^1^,^2^,^3^. Enclosures have been widely used as an efficient management strategy in grassland restoration because they are simple, inexpensive, and maneuverable^1^,^2^,^4^. Enclosure promotes the natural restoration of degraded grassland by eliminating grazing pressure and the restored vegetation then reduces soil erosion; meanwhile, litter increase and root transport rise soil nutrients input. They both help improve the soil nutrient availability^5^,^6^. Besides, enclosure promotes plant height growth that grassland vegetation grows upward to make better use of photo thermal resources. However, the effect of enclosure on grassland closely relates to its duration, as many studies have found that while short-term enclosures can improve biodiversity and productivity, long-term enclosures reduce species diversity in grassland communities^6^,^7^,^8^,^9^,^10^.

The mechanisms underpinning diversity loss have been heavily debated and many studies have focused on the increased soil nutrient due to enclosure, especially N-increasing, based on the established facts that N-enrichment decreased species diversity^11^,^12^,^13^,^14^,^15^. Two hypotheses have been proposed for the decline in species diversity. The assemblage-level thinning hypothesis (or random-loss hypothesis) predicts that N-enrichment reduces the N limitation on grassland productivity, thereby increasing community biomass and individual plant size^11^,^16^. This in turn leads to an increased overall competitive intensity in the community, which causes community-level thinning^17^,^18^ and decreased density, as small individuals of all species randomly die for weaker competitiveness^16^. This hypothesis assumes that individual mortality is equal among species, so rare species would be most vulnerable owing to their small population size, and the death of rare and opportunistic species would reduce the species diversity^17^,^18^. In contrast, the interspecific competitive exclusion hypothesis assumes that mortality is not equal among species, and predicts that subdominant species are suppressed by dominant species, and the competitive exclusion of subdominant species reduces richness^17^,^19^. This hypothesis is often associated with a functional-based hypothesis, i.e., species with traits that confer an advantage under the changed conditions can exclude other species^16^. Almost all competition models emphasize species differences in adaptive strategies, reductions in resource utilization, individual sizes, and growth rates, which competitively rank species and increase the importance of light competition in fertile habitats^20^. Many studies that use these models have concluded that as soil nutrients accumulate^21^, competition switches from belowground to aboveground as light being limiting. The smaller species with poor light acquisition are competitively excluded by dominant species, reducing overall species diversity^22^,^23^,^24^,^25^.

These two hypotheses for explaining diversity loss assume that N availability is an important factor that limits grassland productivity; as N increases, productivity increases, and when primary productivity exceeds a certain threshold, increased community biomass and competition will reduce species diversity^24^,^26^. However, key question remains about how long-term enclosures reduce species diversity. To address this question, we studied an enclosed community for eight years and evaluated the causes of reduced species diversity. Two hypotheses were proposed: (1) in contrast to unenclosed plots, soil N and P (phosphorous) content should increase significantly in enclosure plots, and nutrient limitation effects on enclosure plots should weaken while light competition effects on species diversity increase; and (2) the growth of understory individuals, which were primarily annuals and biennials should be suppressed by vertical light attenuation as community structure changes over eight years. While canopy species, which will intercept more light, should become dominant.

## Results

### Species composition and traits of dominant species

The dominant canopy species in the enclosure quadrats were *Leymus chinensis, Caragana microphylla, Serratula centauroides*, and *Carex duriuscula*, while the dominant understory species were *Cleistogenes squarrosa* and *Carex duriuscula*. There was no obvious community stratification in the clipping quadrats, and the main dominant species were *Poa sphondylodes* Trin., *Cleistogenes squarrosa*, and *Leymus chinensis* (Table 1). Most species in the enclosure and clipping communities were N limited (N: P < 14), except the leguminous plants (*Caragana microphylla, Vicia cracca* L., and *Melissitus ruthenicus* (L.) Latsch.) and *Carex duriuscula* (N:P > 16). Only *Thalictrum squarrosum* Stephan ex Willd. and *Potentilla acaulis* L. in the enclosure community were both N and P limited (N:P ratios of 15.1 and 14.8, respectively). Most common species found in both the enclosure and clipping communities had a lower leaf TN content and N: P ratio in the clipping community (Table 1), indicating that N limitation had been reduced in the enclosure community.

We analyzed the leaf traits of five dominant species in the enclosure and clipping communities and found that traits differed significantly, except for the leaf area of *Serratula centauroides* and the chlorophyll index of *Cleistogenes squarrosa* (Table 2). The height, chlorophyll content, and leaf area of the five species showed a significant increase in the enclosure community, indicating that the species in the enclosure community had better light acquisition abilities (Table 2). Compared to the clipping community, the population densities of two understory species, *Cleistogenes squarrosa* and *Carex duriuscula*, decreased significantly in the enclosure community (by 63% and 55%, respectively), while densities of canopy species *Leymus chinensis* and *Caragana microphylla* increased significantly (Table 2). Two understory species, *Cleistogenes squarrosa* and *Carex duriscula*, remained dominant in the enclosure community.

### N, P content of plant community and soil

**Figure 1:**
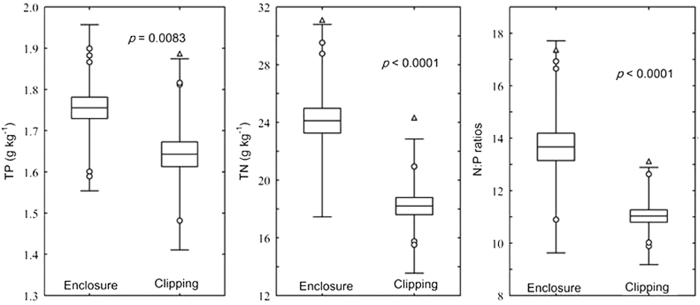
The horizontal line in each box is the mean value, the boxes indicate the Mean ± SE, and the whiskers represent the Mean ± 2*SD. ○Represents Outliers; Δ represents Extremes.

Community N: P ratios revealed that both enclosure and clipping communities experienced N limitation. However, community TN, TP, and N:P ratios of enclosure quadrats were significantly higher than those of clipping quadrats (Fig. 1), so while N was still a limiting factor at a community scale, its limiting effect on community vegetation was decreased in the enclosure community, which was consistent with the results at the species scale.

Although no significant differences were observed in soil TN and TP content between enclosure and clipping quadrats, AN and AvP values in enclosure quadrats were significantly higher than in clipping quadrats (Table 3), and far higher than in grazing quadrats (AN = 106.64 ± 2.42 mg kg^−1^, AvP = 4.34 ± 0.21 mg kg^−1^). The N and P contents of leaves and the AN and AvP contents of soil in enclosure quadrats were all higher than in clipping quadrats, proving that enclosures allowed more N and P to accumulate in the soil. The increased N content in plants might be derived from N accumulation in the soil, which was consistent with our first hypothesis.

### N: P ratios and the role of light on species diversity

**Figure 2:**
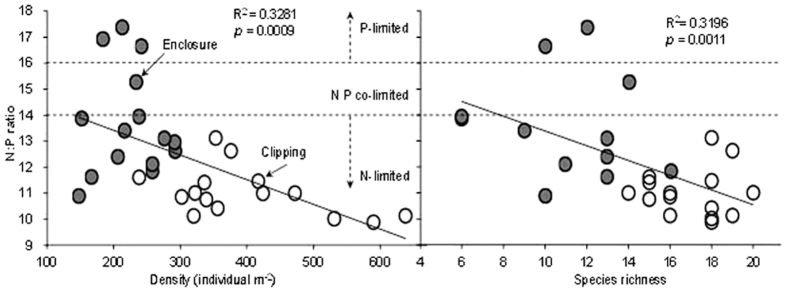
The white circle represents clipping and the gray circle represents enclosure.

N: P ratios of quadrat vegetation showed significant negative correlation with both plant density (*R*^2^ = 0.3281, *P* = 0.0009) and species richness (*R*^2^ = 0.3196, *P* = 0.0011) (Fig. 2), which suggested that with decreased N limitation in the community, plant density and species richness also decreased. The decline in species richness was probably due to N accumulation, and the decline in density might be resulted from the decline in species richness.

**Figure 3:**
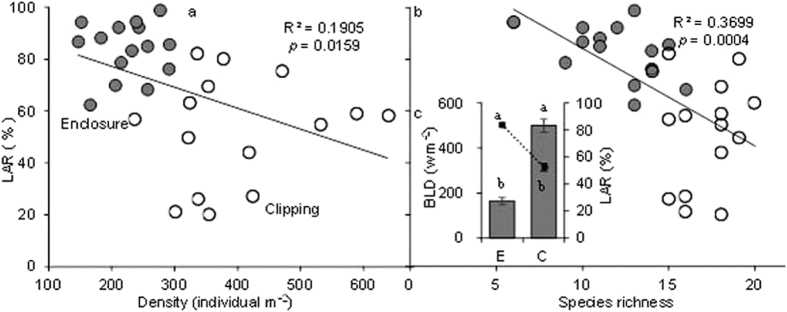
BLD represents bottom light density of community; Gray bars indicate the BLD; black ligature circles represent the light attenuation rate.

The different vertical structures of the enclosure and clipping communities altered light distribution, so the bottom light density (BLD) in the enclosure community was significantly lower than in the clipping community. In addition, the light attenuation rate (LAR) in the enclosure community was significantly higher than in the clipping community; therefore, the low light conditions at the bottom of the enclosure community were not conducive to the survival of plants with poor light acquisition abilities. The LAR had a significant negative correlation with both plant density (*R*^2^ = 0.1905, *P* = 0.0159) and species richness (*R*^2^ = 0.3699, *P* = 0.0004) (Fig. 3), possibly from the death of small individuals and species that could not compete in low-light conditions.

### Comprehensive effects of environmental factors on plant distribution

**Figure 4:**
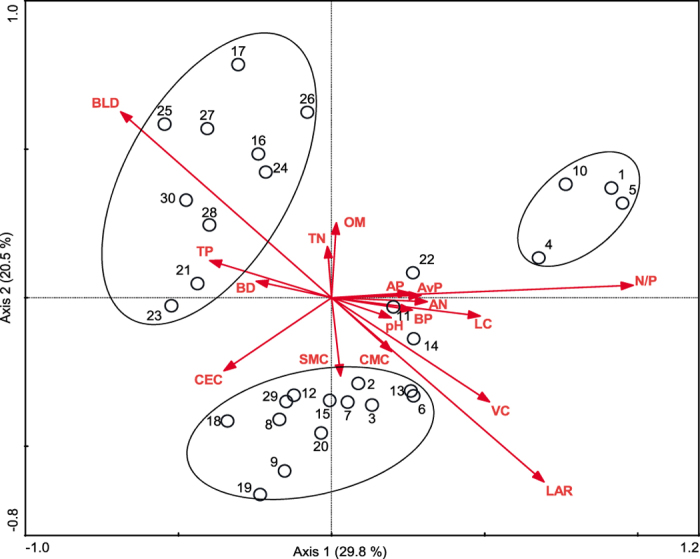
1–15 (circles) represent the enclosure quadrats and the 16–30 represent the clipping quadrats. OM represents Organic matter; TN represents Total N; AN represents Available N; TP represents Total P; AvP represents Available P; SMC represents Soil moisture content; BD represents Bulk density; CMC represents Capillary moisture capacity; CEC represents Cation exchange capacity; BP represents Bulk porosity; AP represents Aeration porosity. LAR represents Light attenuation rate; VC represents Vegetation coverage; LC represents Litter coverage; BLD represents Bottom light density of community.

Species richness was negatively correlated not only with N: P ratios and LAR but also with litter coverage and abiotic factors such as soli TN, AN, and AvP (Table 4), which indicated that nutrient accumulation and increased litter cover might lead to a decline in species richness. No significant correlation was observed between plant density and any environmental factors, so decreased plant density was not related to soil nutrient supply, but it was directly related to the decline in species richness, and the increased competition from dominant species led to decreased community density. The comprehensive analysis of interactions between these environmental factors and vegetation also revealed that plant distribution was not only affected by soil factors such as AvP (Monte Carlo Test, *R*^2^ = 0.063, *P* = 0.049), CEC (*R*^2^ = 0.067, *P* = 0.043), and AN (*R*^2^ = 0.077, *P* = 0.034), but also by light and plant coverage factors such as VC (*R*^2^ = 0.077, *P* = 0.032), BLD (*R*^2^ = 0.158, *P* = 0.002), LC (*R*^2^ = 0.172, *P* = 0.002), LAR (*R*^2^ = 0.159, *P* = 0.002), and plant N:P ratios (*R*^2^ = 0.216, *P* = 0.002). The factors with the maximum effects were N: P ratios, LC, LAR, and BLD (Fig. 4). These results indicated that while enclosure and clipping communities were not significantly different in most soil physicochemical factors, the accumulation of AN and AvP, and changes in light intensity, jointly affected plant growth and distribution. Redundancy analysis (RDA) showed that enclosure and clipping quadrats were mainly distributed at either end of the light axis, and their distribution along a fertility gradient (axis 1) indicated that N limitation was lower in enclosure quadrats. For example, quadrats 1, 4, 5, and 10 were not N limited because they included *Caragana microphylla*, an N-fixing legume. The extent of N limitation in the enclosure quadrats was also less than that in clipping quadrats.

Species richness was analyzed through backward- and forward-deletion multiple linear regression (Table 5) with seven variables (TN, AvP, CEC, AN, LC, BLD, and LAR) that were significantly correlated with plant distribution and species richness. The optimal model of two regression methods contained only two explanatory factors, AN and BLD, and their partial regression coefficients did not differ significantly; therefore, reduced light intensity at the bottom of the community and AN accumulation in the soil were the main causes of the declined species richness.

## Discussion

### What are the dominant traits of the most competitive species?

In this study, species richness and plant density of the grassland community decreased significantly after eight years of enclosure because soil AN and AvP accumulated and BLD decreased. Further analysis was conducted to identify the N, P utilization and light acquiring characteristics of the outcompeted species. Compared with the species in the clipping community, the dominant species in the enclosure community were taller, with greater leaf area and chlorophyll content, which demonstrated better light acquisition ability (Table 2). This indicated that light competition was the driving force behind the success of the dominant species as community height and canopy density increased over years of enclosure. *Caragana microphylla*, a legume, was among the five dominant species in the enclosure communities and was only P limited. As an N-fixing plant, its competitive advantage became increasingly pronounced in the enclosure enclosures, and its leaves had higher N and P contents per unit area and mass (Tables 1 and 2). *Carex duriuscula* was not N limited in both management modes, demonstrating its high N uptake efficiency (Table 1) and adaption to light reduction by increasing leaf area and chlorophyll content in the enclosure treatment (Table 2). The species also had a higher SLA value, but the increased leaf area lowered the N and P contents per unit area. *Cleistogenes squarrosa* was the dominant understory species in both management modes, probably because it is a C_4_ plant with high light utilization efficiency; it also had higher leaf N and P contents per unit area in the enclosure community. *Leymus chinensis* and *Serratula centauroides*, perennial rhizomatous grasses with a clonal reproduction strategy^27^, were taller canopy species with a natural competitive advantage for light, and therefore, were the dominant species in the enclosure community. The most successful dominant species have their respective competitive advantages and light adaptation strategies to outcompete other species in long-term enclosures, and understory species remained dominant through physiological changes that increase their light acquisition capabilities.

Compared with the clipping community, population densities of the dominant understory species decreased significantly, while the dominant canopy species increased significantly, indicating that with increased biomass and intensified competition, individuals from all species did not die randomly during self-thinning in the enclosure community. The understory species with fewer individuals or poor plasticity in a changing light environment probably died first in the community^16^,^27^. This result contradicts the random-loss hypothesis^17^ but supports the competition exclusion hypothesis^16^. Suding *et al*.^16^ summarized 34N addition experiments conducted in North America and found that regardless of the initial density, the risk of extinction increased for N-fixing, perennial, and native plant species after fertilization, with the maximum extinction probability in rare species (>60%), while the minimum was seen in species with the largest population density (only 10%)^16^. Clark and Tilman (2008) also found that rare species were most sensitive to N-addition in their 23 years of field experiments^13^. Our study showed similar results, with extremely low frequencies and minimal population densities of rare species and annual or biennial forbs in the enclosure community enclosed for eight years. In long-term enclosure communities, species richness is also reduced by competitive exclusion, in which dominant species crowd out rare and opportunistic species with lower population densities. This implies that with increased biomass and intensified competition, not all species experienced self-thinning and individual mortality was not equal among species.

### The influence of light competition and N and P enrichment on species diversity

Decreased species diversity seriously threatens nutrient cycling and energy flow in ecosystems^28^. Extensive experiments have suggested that N-addition leads to an asymmetrical supply of resources, which changes the above-ground, below-ground, or the overall competition intensity and reduces species richness^11^,^13^,^15^,^19^,^20^,^25^,^29^,^30^. However, Dickson and Foster (2011) proposed that even when light was not the limiting factor, soil N-increasing would reduce species richness, and multiple factors should be considered to explain the loss of species diversity^14^. In this study, the soil AN, plant N content at the community scale, community height, and community biomass in the enclosure sites were significantly higher than in the clipping sites, indicating that eight years of enclosure did facilitate N accumulation. However, N did not accumulate to the point where it was no longer limiting to the community, as most plant quadrats were still N limited (N:P < 16), but the degree of N limitation in the enclosure community was reduced. Soil AvP also accumulated in the enclosure community (Table 3). Research has shown that as atmospheric N deposition increased, the available N in terrestrial ecosystems has increased significantly (e.g., plant leaf N content of Chinese terrestrial ecosystems has increased 32.8% on average from 1980 to 2000), and a switch from N to P limitation or co-limitation has occurred in grasslands^12^,^13^,^29^. In an experiment using European semi-natural grasslands, Ceulemans *et al*.^12^ found that P-limited grasslands exhibited higher species richness, suggesting that P enrichment could present a greater threat to biodiversity than N enrichment in at least some terrestrial ecosystems, and that N- and P-driven species loss were two independent processes^12^. In our study, although soil AvP was significantly negatively related to species richness, soil TP content (0.41 g kg^−1^) was lower than the average level across China (0.41 g kg^−1^), and most quadrats were N limited (N:P < 14), so we concluded that P limitation and P enrichment were not influential in the enclosure community.

With the increase in biomass, litter accumulation, community height, and canopy density, the amount of light that reached the community bottom decreased. Decreased light in the understory led to the loss of species with greater light requirements or poorer light acquisition capabilities, especially those species whose seeds and seedlings could not complete photomorphogenesis under shaded conditions^1^,^6^,^7^. In our study, species richness showed a significant negative correlation with soil AN, AvP, community N: P ratio and LAR from community surface to bottom, while N accumulation and increased BLD were the main causes of reduction in species richness (Table 5).

## Conclusion

Enclosure had significant effect on plant diversity in Hulunbeier grassland of northern China, as significant decrease in species richness was arisen after eight years of enclosure. A variety of environmental factors had roles in reducing species richness, but AN accumulation and lower BLD were the most significant ones; changes in vertical structure of plant communities as well as competition for light and heat resources had mutual influence on the status and importance of plants in community and ultimately productivity of grassland. Our findings provide not only a more comprehensive understanding of the relationship between enclosure and its effect on degraded grassland restoration but also the mechanism of the effect of plant diversity loss on ecosystem functions. Therefore, from the view of grassland renewal and its sustainable development with animal husbandry, grassland should be properly used after enclosed for a certain period to balance the recovery and utilization of degrade grassland.

## Materials and Methods

### Study site

The experiment was conducted in the Huihe National Nature Reserve Hulunbeier grassland in Inner Mongolia (118°48–119°45′E, 48°10–48°57′N), which has a total area of 3 468 km^2^. The region has a temperate continental monsoon climate. The annual average temperature is –2.4 °C to 2.2 °C. The frost-free period is 100 to 120 d, and the annual average precipitation (2008–2014) was 375.03 mm, 70% of which fell between June and August of each year. This nature reserve is one of the 17 critical areas for biodiversity protection that joined the China Biosphere Reserve Network (CBRN) in 2007.

### Experiment design and plant investigation

A free-grazing pasture with uniform terrain was fenced in June 2008, where we established three sites (each 1 ha). Each site was divided into two approximately equal parts, one of which was clipping. The clipping parts were cut once every year around August 20 (local mowing time), and no grazing or other human disturbance occurred in enclosed parts during the entire experiment period. The clipping and enclosure areas were all fenced to prevent disturbance, and the areas outside the fence was overgrazed with approximately 3.9–4.5 sheep/ha. In mid-August 2015, we established five quadrats (1 m^2^) in typical vegetation in the clipping and enclosure communities at each site, for 15 quadrats of each management mode. The grazed community had a more simple species composition and community structure with significantly less species richness, so we only established five quadrats at two sites, and only soil variables were measured.

In each quadrat, we recorded species richness, vegetation coverage, litter coverage, and community vertical structure, along with the density, coverage, frequency, and height of each species. Coverage was visually estimated. Species height was averaged from 3–5 medium-height individuals in each quadrat. The importance value (*IV*) of each species by management mode (*IV* = relative height + relative coverage + relative density + relative frequency) and by quadrat (*IV* = relative height + relative coverage + relative density) was calculated.

### Soil sampling and analyses

Soil cores (0–15 cm depth) were sampled in a quincunx pattern to make a mixed sample from each quadrat. We collected 15 individual soil samples from the clipping and enclosure sites respectively and 10 samples from the grazing sites. We analyzed 12 variables: pH, total N (TN), available N (AN), soil organic matter (OM), total P (TP), available P (AvP), soil moisture content (SMC), bulk density (BD), capillary moisture capacity (CMC), cation exchange capacity (CEC), bulk porosity (BP), and aeration porosity (AP) (Bao 2005). These variables are a good proxy for processes such as nutrient cycling, biological productivity and building nutrient pools, which are important determinants of ecosystem functioning (e.g., water and soil conservation, soil respiration, and supporting flora and fauna) in dry lands.

### Community light intensity

We measured the surface light intensity (SLD) twice in each quadrat between 10:30–11:30 during every sunny day. Three readings of light intensity at the community bottom (BLD) were also taken 3–5 cm above the soil surface, at three different horizontal positions according to the plant distribution within each quadrat and averaged to calculate the BLD. All measurements were made over five days from August 12–20, 2015, using a LI-250A light meter (LI-COR, Lincoln, Nebraska, USA). Light attenuation rate (LAR) was calculated as:


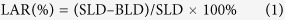


### Leaf N and P content

Intact mature leaves of species were collected from the quadrats and taken to the laboratory after they were scrubbed clean. The leaves of each species were dried to prepare a mixed sample (no less than 5 g) for each site, so each species was represented by three samples from the clipping and enclosure communities. Leaf TN was measured by a CHNS Elemental Analyzer (Elementar, Hanau, Germany) and leaf TP by the Mo-Sb colorimetric method. The average value of the three samples per species was taken as the leaf TN or TP content of that species in the clipping or enclosure communities. The total relative coverage of measured species in the clipping or enclosure communities was greater than 90%.

### Leaf traits of dominant species

In all three sites, 10–20 intact mature leaves of five species (*Carex duriuscula* C.A. Mey., *Cleistogenes squarrosa* (Trin.) Keng, *Serratula centauroides* L., *Leymus chinensis* (Trin.) Tzvelev, and *Caragana microphylla* Lam.), which were the dominant species (according to their *IV* in communities) in both the clipping and enclosure communities (Table 1), were randomly selected to measure their chlorophyll content index (CCI) with a CCM-200 chlorophyll content meter (Opti-Sciences, Hudson, New Hampshire, USA). Leaf fresh weight was determined before leaves were scanned to calculate leaf area with WinFolia 7.0 software (Regent Instruments, Sainte-Foy, Québec, Canada); leaves were then dried to measure leaf dry weight. We calculated the specific leaf area (SLA, cm2·g-1), leaf N content per unit area (N-area, g·cm-2), and leaf P content per unit area (P-area, g·cm-2). The N and P content per unit mass were also calculated (see section Leaf N and P content).

### Ecological stoichiometry characteristics

Vegetation N and P content were calculated by summing leaf N and P content, weighted by species according to their *IV* in a quadrat, and then the community-level N:P ratio was calculated^31^. Ecological stoichiometry indicates that a plant N: P ratio reflects its nutrient supply^32^,^33^: an N: P ratio >16 indicates P limitation at a community level, while an N: P ratio <14 indicates N limitation. At N: P ratios between 14 and 16, either N or P can be limiting or plant growth is co-limited by N and P together^34^,^35^,^36^,^37^. We used these guidelines to evaluate whether a species or the community was N limited.

### Data analyses

Independent samples *t*-tests were conducted to compare soil variables and community N:P ratios between clipping and enclosure communities (*P* = 0.05). The relationships between species richness, community density, and environmental factors were investigated with simple correlation analysis. Redundancy analysis (RDA) was used to assess the relationship between vegetation quadrats and environmental factors. We selected the environmental factors that had a significant correlation with species richness as the explanatory variables and then examined their effect on species richness with a backward- and forward-deletion multiple regression, comparing the relative importance of light and nutrient factors for species richness. Data analyses were conducted with Statistica 8.0 and CANOCO 4.5.

## Additional Information

**How to cite this article:** Li, J. *et al*. Increased soil nutrition and decreased light intensity drive species loss after eight years grassland enclosures. *Sci. Rep.* **7**, 44525; doi: 10.1038/srep44525 (2017).

**Publisher's note:** Springer Nature remains neutral with regard to jurisdictional claims in published maps and institutional affiliations.

## Figures and Tables

**Table 1:** Main dominant species in clipping and enclosure communities.

Site	Species	RH (%)	RC (%)	RA (%)	RF (%)	IV (%)	TN (g kg^−1^)	TP (g kg^−1^)	N:P ratio
Clipping	*Poa sphondylodes*	6.4	5.5	26.1	5.1	43.1	7.1	1.0	7.2
	*Serratula centauroides*	11.4	17.0	5.0	5.9	39.3	16.2	1.5	10.6
	*Cleistogenes squarros*	2.7	14.0	12.6	5.9	35.1	16.8	1.5	11.0
	*Leymus chinensis*	8.5	5.5	9.1	5.9	29.0	18.5	1.6	11.4
	*Carex duriuscula*	4.4	3.1	10.7	5.9	24.2	17.0	1.0	**16**.**9**
	*Galium verum*	4.5	6.6	5.6	4.3	21.0	20.0	2.3	8.9
	*Bupleurum chinensis*	7.8	2.5	2.7	5.1	18.1	22.7	2.3	9.9
	*Caragana microphylla*	2.3	8.8	2.6	2.4	16.1	32.5	1.6	**20**.**8**
	*Koeleria cristata*	3.2	3.3	3.3	4.3	14.1	17.3	1.8	9.4
	*Vicia cracca*	3.1	6.6	1.5	2	13.1	42.4	2.2	**18**.**9**
	*Stipa grandis*	5.3	2.2	1.3	4.3	13.1	12.1	1.3	9.5
	*Potentilla bifurca*	2.8	3.1	2.5	3.5	11.9	28.1	2.8	11.6
	*Artemisia capillaris*	4.6	1.3	1.0	4.7	11.7	21.1	2.3	9.6
	*Belamcanda chinensis*	3.9	1.6	0.5	3.1	9.2	18.9	1.9	10.0
	*Agropyron cristatum*	3.1	0.9	1.6	3.1	8.7	19.1	1.4	13.6
Enclosure	*Leymus chinensis*	13.3	18.3	28.3	8.7	68.6	21.4	1.6	13.4
	*Caragana microphylla*	8.4	24.5	12.1	6.9	51.9	41.1	1.8	**23**.**8**
	*Serratula centauroides*	8.5	18.3	7.9	6.4	41.1	20.0	2.0	10.0
	*Artemisia scoParia*	4.1	17.2	10.1	2.9	34.2	23.2	2.0	11.8
	*Cleistogenes squarros*	4.3	3.5	7.8	8.1	23.8	17.0	1.8	9.7
	*Carex duriuscula*	5.0	1.7	7.4	7.5	21.6	15.2	0.9	**16**.**1**
	*Potentilla bifurca*	5.3	2.3	1.9	5.8	15.3	27.9	2.0	12.0
	*Galium verum*	4.2	1.8	2.9	6.4	15.2	22.6	2.0	11.2
	*Allium ramosum*	5.6	1.2	1.3	5.2	13.3	21.0	2.3	9.3
	*Agropyron cristatum*	3.9	0.8	3.1	3.5	11.3	18.7	2.0	9.5
	*Artemisia frigida*	3.5	0.8	2.4	2.9	9.6	25.2	2.2	11.4
	*Poa sphondylodes*	2.5	0.5	2.7	2.3	7.9	13.9	1.1	12.3
	*Belamcanda chinensis*	3.9	0.7	0.4	2.9	7.8	23.5	1.8	13.0
	*Bupleurum chinensis*	2.4	0.6	0.7	2.9	6.5	25.6	2.4	10.6
	*Cymbaria dahurica*	1.3	0.7	2.1	2.3	6.4	24.5	2.6	9.4

Bold number indicate the N:P ratios > 16. RH - Relative Height; RC - Relative Coverage; RA - Relative Abundance; RF - Relative Frequency; IV - Important Value.

**Table 2:** Traits of common dominant species in clipping and enclosure communities.

Index	Site	Cleistogenes squarros		Carex duriuscula		Leymus chinensis		Serratula centauroides		Caragana microphylla	
Height (cm)	E	17.7	↑	22.23	↑	50.9	↑	44.4	↑	40.1	↑
	C	9.5		15.67		30.0		35.2		20. 7	
Density	E	18.9	↓	19.1	↓	63.6	↑	24.2	—	33.9	↑
	C	50.3		43.1		36.6		20.1		25.8	
CCI	E	18.7	—	16.5	↑	22.9	↑	34.2	↑	50.6	↑
	C	15.6		9.0		16.4		26.7		35.7	
Leaf area (cm^2^)	E	1.7	↑	4.8	↑	13.2	↑	22.9	—	0.9	↑
	C	1.0		1.0		7.6		19.3		0.4	
SLA (cm^2 ^g^−1^)	E	171.18	—	109.04	↑	99.19	—	119.58	↑	114.18	—
	C	186.20		76.20		103.24		85.96		122.82	
N-area (g cm^−2^)	E	107.138	↑	147.324	↓	220.133	—	170.263	↓	364.55	↑
	C	92.808		232.048		187.342		193.439		286.61	
P-area (mg cm^−2^)	E	11.088	↑	9.172	↓	16.414	—	17.064	—	15.700	↑
	C	8.422		13.765		16.506		18.32		13.768	

E – enclosure; C – clipping; CCI - Chlorophyll Content Index; SLA - specific leaf area; N-area - Leaf nitrogen content per unit area; P-area - Leaf phosphorus content per unit area; “—” indicate the values in clipping and enclosure communities had no significant difference, “↑” resprent the values in enclosure communities were greater than clipping communities, and “↓”indicate the values in enclosure communities were less than clipping communities (*P* < 0.05).

**Table 3:** Soil N and P content in enclosure and clipping communities.

Soil index	n	Site	Mean ± SE	SD	*t*	*P*
TN (g.kg^−2^)	30	E	2.07 ± 0.07	0.27	0.18	0.855
		C	2.05 ± 0.06	0.23		
TP (g.kg^−2^)	30	E	0.41 ± 0.01	0.03	−1.85	0.075
		C	0.43 ± 0.01	0.03		
AN (mg.kg^−2^)	30	E	148.82 ± 3.68	14.24	2.35	0.026
		C	137.06 ± 3.40	13.16		
AvP (mg.kg^−2^)	30	E	5.55 ± 0.41	1.58	2.40	0.024
		C	4.50 ± 0.13	0.48		

**Table 4:** Correlation between environment factors and species richness and plant density.

Species diversity	Environment factor										
	pH	OM	TN	TP	AN	AvP	CEC	BD	CMC	BP	AP
Richness	−0.06	−0.33	−0.38*	0.05	−0.59***	−0.39*	0.05	0.14	−0.33	−0.16	−0.17
Density	−0.16	0.10	0.15	0.26	−0.20	−0.02	−0.03	0.03	−0.09	−0.03	0.01

Figures in table were the correlation coefficient; *P < 0.05; ***P < 0.001.

**Table 5:** Best-fitting models of richness by backward- and forward-deletion multiple regression.

Predictor variable	Unstandardized Coefficients	Standardized Coefficients	*t*	*P*	*R*^*2*^
Intercept	26.853		4.916	.006	0.522
BLD	0.007	0.455	3.168	.000	
AN	−0.104	−0.417	−2.903	.013	

Explanatory variables included TN, AvP, CEC, AN, LC, BLD and LAR.
